# Unexpected: an interpretive description of parental traumas’ associated with preterm birth

**DOI:** 10.1186/1471-2393-13-S1-S13

**Published:** 2013-01-31

**Authors:** Gerri C Lasiuk, Thea Comeau, Christine Newburn-Cook

**Affiliations:** 1 Faculty of Nursing, Level 3, Edmonton Clinic Health Academy, 11405-87 Avenue, University of Alberta, Edmonton Alberta, Canada T6G 1C9; 2 Department of Educational and Counselling Psychology, McGill University, 3700 McTavish Street, Montréal, Québec, Canada H3A 1Y2

## Abstract

**Background:**

Preterm birth (PTB) places a considerable emotional, psychological, and financial burden on parents, families, health care resources, and society as a whole. Efforts to estimate these costs have typically considered the direct medical costs of the initial hospital and outpatient follow-up care but have not considered non-financial costs associated with PTB such as adverse psychosocial and emotional effects, family disruption, strain on relationships, alterations in self-esteem, and deterioration in physical and mental health. The aim of this inquiry is to understand parents’ experience of PTB to inform the design of subsequent studies of the direct and indirect cost of PTB. The study highlights the traumatic nature of having a child born preterm and discusses implications for clinical care and further research.

**Method:**

Through interviews and focus groups, this interpretive descriptive study explored parents’ experiences of PTB. The interviews were audiotaped, transcribed, and analyzed for themes. Analysis was ongoing throughout the study and in subsequent interviews, parents were asked to reflect and elaborate on the emerging themes as they were identified.

**Results:**

PTB is a traumatic event that shattered parents’ taken-for-granted expectations of parenthood. For parents in our study, the trauma they experienced was not related to infant characteristics (e.g., gestational age, birth weight, Apgar scores, or length of stay in the NICU), but rather to prolonged uncertainty, lack of agency, disruptions in meaning systems, and alterations in parental role expectations. Our findings help to explain why things like breast feeding, kangaroo care, and family centered practices are so meaningful to parents in the NICU. As well as helping to (re)construct their role as parents, these activities afford parents a sense of agency, thereby moderating their own helplessness.

**Conclusion:**

These findings underscore the traumatic nature and resultant psychological distress related to PTB. Obstetrical and neonatal healthcare providers need to be educated about the symptoms of Acute Stress Disorder (ASD) and Posttraumatic Stress Disorder (PTSD) to better understand and support parents’ efforts to adapt and to make appropriate referrals if problems develop. Longitudinal economic studies must consider the psychosocial implications of PTB to in order to determine the total related costs.

## Background

The birth of a new baby is an exciting and joyful time for most families. The awesome responsibility of caring for a tiny and helpless being can also make it a time of uncertainty and worry. Very quickly after their baby is born, watchful parents become exquisitely attuned to their child’s preferences, personality, and daily rhythms. Small changes in bodily functions or routine can become a big concern, even in healthy infants. *Why won’t she eat? Why is he always hungry? She never naps! I can hardly even wake him up even to eat! What does it mean when ... Is it normal to ...?* This stress and uncertainty is amplified when a baby is born preterm.

A preterm baby is one born before 37 weeks gestation. More specifically, infants born before 28 weeks gestation are considered extremely preterm; those born 28 to <32 weeks gestation are very preterm; and moderate to late preterm infants are born 32 to <37 weeks gestation. Despite advances in health care, preterm birth (PTB) is the leading cause of infant mortality, pediatric morbidity, and long-term disability [1]. In 2010, an estimated 15 million babies around the globe (>1 in 10) were born prematurely [2]. More than 1 million of these babies died as a direct result of their prematurity, making PTB the second-leading cause of death among preschoolers [3]. Although the causes of PTB are not fully understood, the short-term and long-term outcomes are well- documented in the medical literature. Preterm infants are at increased risk for a range of adverse outcomes, including retinopathy of prematurity [4], respiratory distress syndrome and bronchopulmonary dysplasia [5], brain injury [6], necrotizing enterocolitis [7], and, neonatal sepsis [8]. Long-term sequelae include the risk for motor and sensory impairment, learning problems and neurocognitive impairment, and behavioural problems [9-14]. PTB and low birth weight are also associated with lifelong chronic conditions such as dyslipidemia and hypertension [15,16].

Although there have been many advancements in neonatology, technologies and treatment, the incidence of acute and chronic sequelae of PTB have not decreased [17-19]. These trends result in rapidly rising health care expenditures and place significant emotional and financial burdens on families, finite health care resources, and society as a whole [20,21]. Newburn-Cook and her colleagues found the direct medical costs of the initial hospital stay for singleton preterm births to be $20 million (CAD) [22]. The same authors also reported that the costs per infant for the initial hospital admission and the direct medical costs for the first seven years of an infant’s life are inversely associated with gestational age. A limitation of the study was its inability to measure the indirect costs associated with PTB such as adverse psychosocial and emotional effects, family disruption, strain on relationships, alterations in self-esteem, deterioration in general health, mental health, and domestic violence [23,24]. To rectify this, the 2007 Alberta *Consensus Conference on How to Prevent Preterm Birth* called for “... research on the economic impact of preterm birth that includes a more comprehensive analysis of both direct and indirect medical costs... to better evaluate the cost-effectiveness of new policies or interventions” [22] p4. With this in mind, the aim of this inquiry is to understand parents’ experience of PTB to inform the design of subsequent studies of the total direct and indirect cost of PTB. This article highlights the traumatic nature of having a child born preterm and discusses implications for clinical care and further research.

## Methods

We employed interpretive description (ID) to inform an understanding of parents’ experience of PTB. Developed by Thorne and colleagues [25-27] as a way to generate clinically relevant knowledge for health disciplines, ID is a qualitative methodology guided by the ontological and epistemological traditions of human science research [28]. Like other interpretive methodologies, ID rejects the notion of a single, immutable reality that is wholly accessible through empirical methods. Rather, it assumes the existence of multiple realit*ies*, which are context-bound, experientially based, and intersubjectively constructed through social interaction [29,30].

As a human science research method, ID represents a blending of hermeneutic practices with qualitative empirical methods. Through hermeneutic practices, researchers aim to describe and interpret the lived world as experienced in everyday situations and relationships. The focus is on the immediacy of human experience in order to produce qualitative portrayals of a particular phenomenon or event—in this case the parental experience of having and caring for a preterm infant. A central feature of hermeneutic practice is the use of qualitative empirical methods to gather lived experience descriptions, from which underlying patterns and structures are drawn. ID acknowledges the experiential, theoretical, and practical knowledge that researchers and participants bring to a project. Knowledge development is viewed as a continuous, inductive process of (re)negotiating shared understandings about phenomena of common interest. Using constant comparative methods, researchers and participants co-construct an ordered, coherent, and persuasive narrative that can inform clinical practice [26-28]. That said, the goal of ID is not representative sampling in order to generalize findings to a population of interest, but instead to explore, describe, and explicate possible human experience.

## Ethical oversight

Ethics approval was obtained from the University of Alberta Health Research Ethics Board and Alberta Health Services Operational Approvals were obtained through the Northern Alberta Clinical Trials and Research Centre (NACTRC). All participants provided written informed consent.

## Sample

We used purposive sampling to recruit participants from in and around a large Western Canadian city. This non-probability sampling strategy is common in qualitative research and rests on the premise that researchers’ knowledge of the topic area enables them to identify individuals who can contribute meaningfully to the aims of a study [31]. Individuals were eligible to participate if they (1) were parents/primary caregivers of infants/children born preterm between January 2003 and February 1, 2009; (2) health professionals who worked with preterm infants/children and their parents/primary caregivers; (3) spoke and read English; and (4) provided written informed consent. All volunteers who met these inclusion criteria were enrolled in the study and the information they provided was pooled for analysis (i.e., we made no effort to parse our analysis according to demographics, infant gestational age, length of hospital stay, Apgar score, diagnoses, etc.).

Through posters in health-serving agencies, print ads, and word-of-mouth, we invited potential participants to contact the first author (GL), who provided additional information about the study and answered questions. Individuals who consented to participate were assured that they could withdraw at any time without explanation or penalty, although none did. Participants were offered an honorarium of $20 CAD to compensate them for incidental expenses; several individuals declined the honorarium and asked that the money be donated to a local neonatal intensive care unit (NICU).

Fourteen parents (11 women and 3 men) participated in face-to-face or telephone interviews and seven parents (4 women and 3 men) took part in two focus groups to discuss and refine the study findings. Four of the parents who participated in the focus groups also participated in an interview. Parents ranged in age, education, and socioeconomic backgrounds; their infants were born between 25 and 36 weeks gestation. Three sets of parents had more than one singleton child born preterm, but none experienced an infant death. We also interviewed five healthcare providers who worked with preterm infants/children and their families about their observations of parents’ experiences. In total, 22 individuals (17 parents and 5 health professionals) contributed information to the study. Data from the health professionals provided contextual information about local services and alerted us to potential issues of concern to parents; we did not include it in our analysis.

## Data collection and analysis

In keeping with ID, data collection and analysis proceeded concurrently, with each iteratively informing the other. Data were gathered through semi-structured conversational interviews conducted by the first two authors (GL and TC), both of whom have extensive counselling experience (see additional file 1 for the interview guide). Participants were interviewed once or twice for 45 to 90 minutes (in person or via telephone) and all were contacted again the following day to ensure that the interview had not engendered any distress and to offer a referral to a mental health counsellor if necessary. Most of the parents expressed appreciation for the opportunity to talk about their experience and none required counselling. The conversational interviews were audio-recorded, transcribed, and reviewed to ensure clarity and accuracy of transcription. Participants were assigned a unique code number and all other identifying information was altered to protect privacy.

Holistic and line-by-line readings of transcripts were performed for thematic exploration of lived experience descriptions. We began by immersing ourselves in the transcripts and digital recordings to identify themes and patterns and moved on to open coding, during which each meaningful segment of text was assigned a conceptual code. As the codes became saturated, we moved on to pattern coding whereby specific dimensions of parents’ experience of preterm birth were clustered into recurring themes. We asked new participants to reflect and elaborate on these themes as they were identified. Throughout the study, we met regularly to compare and discuss our analyses and to ask, “What is going on here?” and “What are we learning about this?” in an effort to move from the particular to the whole. As the analysis progressed, we endeavoured to articulate a holistic understanding of parents’ experience of preterm birth.

To ensure methodological rigour we provide explicit and detailed information about our epistemological stance and methods; maintained an audit trail; and grounded our findings in the data [32,33]. In addition, we presented the final themes to two focus groups of parents of preterm infants/children who confirmed that the identified themes reflected their experience.

## Results and discussion

None of the parents in this study expected that their babies would be born preterm and when it occurred, it shattered their taken-for-granted expectations of parenthood. With little warning, they were catapulted into the alien world of the NICU, where frightened and helpless, they were forced to rely on strangers to safeguard the survival of their tiny infants. In the early days after their baby’s birth, the normalcy of parents’ everyday life faded into the background and their only concern was their babies’ precarious survival. They existed in a liminal state, in which their babies had been born and yet not quite born and they were parents, but not really able to parent. Uncertainty remained high and even small changes in their babies’ health status could trigger a new crisis. Parents’ ability to adapt to their new and shifting reality was facilitated or constrained by their perceptions of their infant’s survival/well-being; the suddenness, course, and duration of uncertainty about their infant’s survival/well-being; personal and family resources; quality of their relationships with health care professionals; and availability of social support.

## Shattered expectations

For the parents in this study, becoming pregnant was a highly anticipated event that set them on a journey from woman to mother, man to father, and from couple to family. As individuals and together, they prepared themselves psychologically and emotionally for their new roles and began to build a relationship with their unborn child. For women, in particular, the growing being inside of them was always *baby* and never merely a zygote, tissue, or a fetus and they took seriously the responsibility to safeguard his/her life and well-being. All women had regular prenatal care and actively sought out any available information about pregnancy, childbirth, and motherhood. Some referred to popular books such as *What to Expect When You are Expecting *[34], which describes a baby’s week-by-week development and explicitly reinforced their expectations of a normal pregnancy and the delivery of a healthy, happy, full term infant.

The onset of early labour and the premature birth of their baby was a sudden and unexpected event that shattered the parents’ expectations ‘how things should be’, leaving them frightened and disoriented.

I was completely shocked… 23 weeks was too soon, too soon … I knew that at 23 weeks there was no chance of survival… I was told, uhm, they don’t even try to save babies ‘til 25 weeks because of obviously long-term and short-term problems… (Participant, Mother)

… you shut down essentially… we hit a point where both of us were just in total and utter shock. We didn’t know what to do, what to say…it’s like you’re in this really messed up dream and everything’s moving faster than you can comprehend it. (Participant, Father)

This distress was apparent even among the women who experienced health problems during their pregnancies and those who knew that their unborn children would likely have congenital health problems. This suggests that having a cognitive understanding about the potential for having a PTB may not alter expectations of a ‘normal delivery’, nor moderate parental shock when it does occur.

For one woman, whose amniotic sac ruptured during her second trimester, being on bed rest for 10 weeks taxed the family’s resources and constantly reminded them about the possibility that their baby girl would be born prematurely. During that time, the woman’s husband continued to operate the family business, managed the household, and shared the care of the couple’s 5-year old twins with their maternal grandmother, who drove five hours every other week to help out. At their weekly appointments, the woman’s obstetrician apprised the couple of the probability of their baby’s survival and potential for having various disabilities if she was born during the upcoming week. While the woman appreciated her doctor’s honesty, she still managed to remain hopeful.

… he never painted this rosy picture for me in a way that he was guaranteeing me a good outcome, but he made it sound like [having a full-term pregnancy] was … doable. (Participant, Mother)

Despite these constant reminders of possible a PTB, when she went into labour at 33 weeks gestation, she struggled to understand and accept it:

[The nurse said] ‘We’re just gonna let nature take its course and when the baby is born you can hold her until she dies’. She didn’t use the word “dies” but that’s basically what she was saying… I thought like, this is unbelievable, like how could this be happening? What did I do, what did my husband do,…why is this happening? (Participant, Mother)

After their babies were born, the parents were further alarmed by the urgent activity of delivery room staff and the immediate transfer of their babies to the NICU.

[It was] all very surreal… it all happened very fast and very slow at the same time … he needed some help breathing… [and] he got taken away pretty quickly… I actually didn’t get to see him. I don’t remember seeing him. (Participant, Mother)

… they let me kiss his little head before they took him off up to the NICU … it was just a big blur… kinda an out-of-body experience… (Participant, Mother)

… she wasn’t breathing very well, so they took her away, which was horrifying because, you know, you just have this baby and your intention is to hold it and bond with it afterwards and they take it away. (Participant, Mother)

Fathers experienced additional anguish when they had to choose between remaining with their partners or following their infants to the NICU. One man, who waited in an anteroom during his wife’s emergency Caesarean section told us,

I was freaked right out...and then they come out with these kids and [told me] ‘OK, let’s go!’ So now I’m up in the NICU and my wife’s in this other room… there was a lot of back and forth… I think I went 36 hours without sleep. (Participant, Father)

For some of the mothers, the stress of PTB was compounded by feelings of guilt and inadequacy, which eroded their trust in their bodies, their womanhood, and their abilities as mothers.

… I also felt like that it was all my fault and … I felt a grieving that I couldn’t mother him the way that I would if we coulda taken him home that, the next day… I wasn’t doing my job properly and [I felt] inadequate knowing that - as a woman- I was not capable of doing it the way everyone should do it. (Participant, Mother)

I remember the first time going [into the NICU] … I wanted to throw up, not from being nauseous but because all of a sudden I just felt this overwhelming responsibility, like I did that to her. … (Participant, Mother)

## Helplessness and horror: the trauma of PTB

The extreme stress of PTB was compounded by the strange and alien environment of the NICU, where parents did not speak the language and where every beep and light signaled another crisis. With their dreams of a normal birth and robust infant wrenched from them, they were confronted with impossibly tiny infants who were being kept alive “betwixt the womb and the world” [35] p326 by banks of ominous looking machines. Uncertainty was high and the parents felt horrified and helpless.

… it was horrifying… a healthy baby kinda looks like a frog… their legs are pulled up to their stomach and their arms are bent – [my baby] was just flat and long [because] she had no muscle tone. It was horrible (Participant, Mother)

… my brother-in-law went up with me to the NICU and… it was good to have him there because I pretty much fell on my knees… it was very, very scary… (Participant, Father)

… he was so skinny! I’m like, ‘That doesn’t look like a baby!’ How am I gonna’ take this kid home and take care of him…I don’t know if I’m saying it right – but how are we supposed to take this kid home and get him strong, you know. I don’t know if I can handle [it], he looked so frail! (Participant, Father)

Despite being disoriented and overwhelmed, the parents gradually adapted to the NICU and began to negotiate what it meant to be a parent and *how* to parent in a strange and frightening environment.

As the study progressed, it became obvious that when the parents’ spoke about the stress associated with having a preterm infant, their experience exceeded usual or everyday levels of stress. Our interviews revealed that the threat of an imminent PTB precipitated a crisis for them which, according to Mitchell [36], is a reaction to an acute stimulus or demand (stressor), characterised by disturbances in perception, emotion, and thinking; a failure of usual coping mechanisms; and impairments in function. Like other crises, PTB disrupts parents’ fundamental assumptions about their selves, the world, and their place in it. These fundamental assumptions are termed differently by different people. The psychiatrist, John Bowlby [37,38] referred to them as working models that we construct about our self and the world and which give meaning to our perceptions. Parkes [39] p132 used the term ‘assumptive world’ to describe the “strongly held set of assumptions about the world and the self which is confidently maintained and used as a means of recognizing, planning and acting …” Similarly, Epstein [40] believed that each of us has a personal theory of reality, which includes both a self-theory and a world-theory. This “personal theory of reality does not exist in conscious awareness, but is a preconscious conceptual system that automatically structures a person’s experiences and directs his or her behavior” [40] p65. For Janoff-Bulman [41]p5, our fundamental assumptions are the “bedrock of our conceptual system” and the foundation of our most basic beliefs about our self, the world, and the relationship between the two. Because they are abstract and - like the air we breathe - ubiquitous, we are largely unaware of them and unlikely to question them. They simply exist in our minds as ‘the way things are’. Janoff-Bulman further contends that unless confronted with evidence to the contrary, most of us share some version of the following three fundamental assumptions – “The world is benevolent. The world is meaningful. The self is worthy” [41]p 6. PTB challenges these assumptions, compelling parents to reconstruct their systems of meaning in order to make sense of their new reality.

The magnitude of the crisis of PTB is consistent with the American Psychiatric Association’s (APA) [42] definition of a trauma. The APA defines a traumatic event as one that threatens an individual’s life or integrity (or the life/integrity of a loved one) and evokes a sense of helplessness and horror. PTB was traumatic for parents in this study as it altered their perceptions, strained their coping resources, and triggered a range of physical, emotional, and behavioral responses including fear, anxiety, grief, depression, changes in appetite and sleep patterns, and social withdrawal. When asked, the parents in the study all agreed that the term stress’ does not fully reflect their experience, but that the term ‘trauma’ does.

## Focus on the infant’s precarious health

Depending on their degree of prematurity, the prognosis of some of the infants was guarded especially during their early days in the NICU. A few had serious health problems, with ominous sounding names like respiratory distress syndrome, necrotizing enterocolitis, Tetralogy of Fallot, and Cerebral Palsy. Most of the infants underwent a number of diagnostic and/or surgical procedures, some of which required transfers to other hospitals. As their parents struggled to take it all in, the day-to-day concerns of normal life faded into the background and their only concern was their child’s survival.

… I spent all of my time at the hospital. I mean when I thought [my baby] was dying… I had this overwhelming feeling like, this is where I have to be. I have to be with my daughter because if she dies, I mean … however long [she was ] on this earth, [she should] be with her mom, right? With her family. (Participant, Mother)

… things that we thought mattered before, you find out really don’t matter … like financial costs...When we did our income tax that year [we realized that we] spent a lotta money, but you don’t care… you just don’t worry about that stuff because your head’s somewhere else. (Participant, Father)

The first hours and days after their baby’s birth were particularly difficult. They are strangers in a foreign and frightening environment, where they do not know the terrain and do not speak the language. In shock and terrified, their only concern was their baby’s survival. This concern fuelled parental vigilance and a strong desire to remain with their infant. Despite feeling helpless to ‘do anything’ the parents spent long hours in the NICU, often at some cost to their own wellbeing.

… nothing else, nothing for me mattered at all; I mean I barely remembered to eat (Participant, Mother)

… the whole routine was, was hard. It became quite a job, to be honest, get up in the morning, have breakfast, go to the hospital for 12, 14 hours, as long as you could and then come back and do it all over again, 7 days a week… he was in [the NICU] for 54 days … it was draining. (Participant, Father)

In order to stay at the hospital, parents reorganized almost all other aspects of their lives and when it was available, they drew assistance from extended family and friends. Some of the women had to quickly hand-off unfinished work to co-workers and organize an early maternity leave; those who had children scrambled to find care for them. Likewise, most of the men arranged some time off work but as the sole wage earners, many of them felt the burden of ensuring the family’s financial stability. The financial strain of having a preterm infant was particularly salient for the women and men who were self-employed and those whose income was already reduced because of other circumstances.

…it was a huge financial cost for me and emotional cost too because I would be at the hospital cafeteria with my laptop trying to deal with work stuff… my dad ended up flying from [another country] and helping me with my business… the company lost quite a bit of money in those months but, you know, again it just was what it was and obviously, it wasn’t a priority, right? I mean the health of our of my son and our family was a much bigger priority than the business. (Participant, Mother)

All of a sudden we have a baby and [my wife] went on mat leave 3 months before expected and I was on reduced pay, so that the financial situation was compounded... (Participant, Father)

Other sources of financial stress were related to the transportation and parking costs, the cost of eating at the hospital, childcare costs for older children, and to purchasing special equipment. Some couples borrowed money to help them cover these extra expenses, while others received money and other types of assistance from family and other support groups.

… you just figure well, if we go into debt a little bit now we’ll just have to do some catching up later, uhm, because you know this is just what we need to do ... (Participant, Mother)

… we were on a tight budget already… it was always in the back of my mind, it’s like ‘OK, I have to go get something to eat and I only have a limited funds, what’s the cheapest thing I can get?’ … Eventually our church… collected a fund for us (Participant, Father)

Grandparents, in particular, also bore emotional and financial costs of PTB.

… both grandmothers took some time off of work to help. (Participant, Father)

… if I didn’t have [my mom] I don’t know what I would have done… she brought me food to the hospital so that I wouldn’t have to eat the hospital food... [and] they drove us everywhere, even after [our daughter] came out of the hospital (Participant, Mother)

## Prolonged uncertainty: cycles of crisis and adaptation

As their infants’ health stabilized, the parents slowly regained their equilibrium, began to rebuild their meaning systems, and gradually adapted to their new, but precarious reality – a reality that was easily disrupted by even small changes in their infant’s condition or the NICU routine.

… having a baby in, in, in the NICU is that it really feels like a bit of a rollercoaster… at one moment in time it looks like things are getting better, and then your baby has a really bad night or your baby has a bad few hours ... [it’s] two steps forward [and] one step back (Participant, Mother)

… every day was different, you couldn’t, you couldn’t go in there expecting the same thing that happened yesterday or even a, a good step, `cause if you, if you went in expecting a weight gain and there was a weight loss it was just a complete downfall… you had to kind of pick yourself up again … (Participant, Father)

This prolonged uncertainty about their infant’s survival was the crux of the trauma of PTB and coupled with their lack of agency, it kept the parents in a heightened state of arousal. From the stress literature we know that the perception of a threat to one’s own life or the life of a loved on triggers an automatic, total-body response that Cannon [43] dubbed the fight-or-flight response. Within seconds, cascades of hormones and neurotransmitters course through the body marshalling every cell into action [44]. When the stressor abates or an individual is able to escape from it, the stress response systems return to their resting states. However, as we heard from the parents in this study, when the health/well-being of their infant is tentative, the threat remains omnipresent.

## Fostering adaptation

Parents’ ability to adapt to their new reality was influenced by three main factors; (1) their personal and couple resources; (2) the quality of their relationships with the NICU staff; and (3) the presence of social and functional support. These factors appear to buffer the severity of the trauma as parents cope with the precarious health of their infant.

Personal and couple resources included the parents’ constitutional and psychological characteristics, past learning, problem-solving strategies, ability to manage their emotions, and the quality of their relationship. Although the Canadian system of publically funded healthcare buffered the economic impact of having a preterm infant, economic factors contributed to parents’ stress, particularly for those who were self-employed, lived a distance from a tertiary care hospital, or whose infant was hospitalized for an extended period of time.

In the beginning it was very, very taxing and very stressful and very hard on us and, uh, tested a lot of things about our, our marriage and finances and everything (Participant, Father)

… my wife and I have been married 6 years and we really hadn’t gone through anything... a hardship together … so to go through something together that affects you so much emotionally, uhm, and you kinda learn how to, how to support each other… now I think we know each other better… (Participant, Father)

Relationships with NICU Staff also influenced parents’ adaptive capabilities – not surprisingly, positive relationships enhanced adaptation, while negative relationships contributed to parental stress.

[My doctor] was really good because he was a great advocate for us … some of the, the nurses really wanted to take over and, uhm, do what they felt was best. I was really lucky in that this doctor was a huge advocate for us. (Participant, Mother)

Yeah, it was huge to, you know, when I’d walk in there in the morning and see a nurse that I knew [and] I would just feel better about the day just knowing like, ‘OK you’re, you’re a good nurse’ … I’d be nervous when she had a new one but she would just be fabulous too. (Participant, Mother)

I wouldn’t even look at the nurses, I was so mad … the social worker ended up meeting me … I just went like completely ballistic because [sighs] again… lack of control … like I have no say over what’s going on. … (Participant, Mother)

Not surprisingly, parents also experienced social support as protective. Those who could rely on friends, faith communities, or other social relationships (including other parents in the NICU parents) for emotional, physical, and psychological support fared better than those who did not have these.

… both of us have lots of family here so we had a ton of offers of support and, uh, you know lots of people were nice and brought us lasagnas and … aunts coming over here during the day and tidying up the house… (Participant, Father)

I sent [my children] to stay with my husband’s grandparents … I cried, I felt like such a bad mom, you know, having to send them away [but] I knew my twins would be OK and they’d be fine with other family ... (Participant, Mother)

From this inquiry, it is clear that like other health crises and life-threatening events [45,46], the experience of having an infant born preterm is a very stressful - even traumatic - event for most parents. Traumatic events sorely challenge an individual’s coping resources and evoke a range of physical, emotional, and behavioral responses such as fear, anxiety, grief, depression, changes in appetite and sleep patterns, and social withdrawal.

Recently, clinicians and researchers have applied the constructs of acute stress disorder (ASD) and posttraumatic stress disorder (PTSD) to explain parents’ experience of PTB. Diagnostic criteria for ASD [37] include exposure to a traumatic event, which an individual perceives as life threatening and to which he/she responded to with intense fear, helplessness, or horror. In the wake of the distressing event, the individual must also experience increased autonomic arousal, dissociative symptoms (e.g., emotional numbing, depersonalization, or amnesia); and re-experiencing of the event through intrusive thoughts, dreams, or ‘flashbacks’). These disturbances cause significant impairment in social, occupational, or other important areas of functioning and if these symptoms last longer than one month, the individual meets diagnostic criteria for PTSD.

A recent systematic review of research revealed that posttraumatic symptomatology is not uncommon in parents or primary caregivers of premature infants [47]. One study in that review found that 67% of mothers with preterm infants (vs. 6% of a control group of mothers of full term infants) exhibited PTSD symptoms [48]. Similarly, Holditch-Davis [49] and her team found that all of the mothers in their study had at least one posttraumatic symptom, 12 had two symptoms, and 16 had three symptoms, post birth. Kersting’s group [50] reported that compared with mothers of healthy term infants, mothers of very premature infants showed significantly (p < .05) higher rates of traumatic symptoms at 1–3 days, 14 days, 6 months and 14 months post birth . Finally, Wereszczak et al [51] found that even 3 years after a PTB, caregivers reported vivid memories related to their infant’s appearance, behaviour, pain, procedures, illness severity.

More recently, an Alberta study [52] employed a prospective cohort within-subjects design to explore the number and severity of ASD symptoms in parents of preterm infants at 7 - 10 days and one month post-PTB. The authors found that 25% of respondents (28% of mothers and 17% of fathers) met diagnostic criteria for ASD at both measurement times. The authors also reported significant depression scores among 43% of the mothers at 7 - 10 days and in 35% of mothers at one month and among 33% and 17% of the fathers respectively. This latter finding is noteworthy because maternal depression has implications for the mother’s psychological well-being and the infant’s cognitive and emotional development. Prior research demonstrates that the infants of depressed mothers are at greater risk for developmental disturbances [53-56], which may compound the effects of prematurity. In a similar study, Shaw et al. [56] found that almost half of the mothers, but none of the fathers, in their study met all diagnostic criteria for ASD. Those authors suggest that women’s increased levels of distress may be related to their greater psychological involvement with their infants. Others [57,58] attribute this to gendered differences in coping styles, whereby men cope by discounting the severity of the problem; deny or minimize their own emotional responses; and focusing their energies on supporting their partners during the NICU hospitalization.

Other studies demonstrate that symptoms of parental traumatization after PTB can continue at 6 and 18 month after the infant’s discharge from hospital [48] and take the form of intrusive memories and efforts to avoid reminders of their experiences. In other work [59] that compared mothers of premature and full-term infants, the former had significantly more symptoms of intrusion, avoidance, and hyperarousal.

## Conclusions

Parents in this qualitative study highlight disruptions in meaning systems, prolonged uncertainty, and lack of agency as factors that contribute to the psychological trauma associated with having a preterm infant. This resonates with Shaw et al.’s finding that parental trauma is less related to infant characteristics (e.g., gestational age, birth weight, Apgar scores, or length of stay in the NICU) than it is to alterations in the parental role expectations. This included being unable to help, hold, or care for their baby; protect him/her from pain; or share the baby with other family members. We also speculate that this disruption in parental role expectations are part of global disturbances in their fundamental assumptions about their selves as worthy and agent and the world as safe and predictable [41]. Our findings also help to explain why things like breast feeding, kangaroo care, and family centered practices are so meaningful to parents with an infant in NICU. As well as helping to (re)construct their role as parents, these activities gave participants a sense of agency - of doing something tangible for their baby - thereby moderating their own sense of helplessness.

Given the nature of qualitative research, the findings and conclusions of this study are not generalizable to all parents of preterm infants. The sample size is small and unrepresentative and the data is all self-report. In considering the findings, it is also important to keep in mind that traumatized individuals may not participate in research related to PTB in order to avoid reminders of past experience. That being said, the findings underscore the importance of early identification and treatment of psychological distress related to PTB. Obstetrical and neonatal healthcare providers need to be educated about the symptoms of ASD and PTSD to better understand and support parents’ efforts to adapt and to make appropriate referrals if problems develop. The findings also endorse the importance support from healthcare providers in assisting parents to assume a parental role. In addition, this inquiry underscore the need for ongoing efforts to identify parental psychological distress and to develop effective screening and trauma-informed intervention strategies to help parents at risk for psychological distress in the wake of PTB.

Despite the trauma associated with having and caring for a preterm infant, all of the participants in our study stated that they would be willing to participate in long-term, economic studies about the direct and indirect costs of PTB with the some provisions:

### Timing

Researchers should approach potential participants in-person, while their baby is still in the hospital and after their baby’s health has stabilized. Parents emphasized the importance of developing and sustaining personal and trusting relationships with members of the research team.

### Altruism and meaning-making

The participants in our study told us that they had very few opportunities to tell their stories of having a preterm infant and they welcome opportunities to participate in research that might make a difference for other parents. The possibility of improving the healthcare experience of others (altruism) helped parents make sense of their own experience and of having something positive come out of something that was difficult. This has implications for recruitment because NICU personnel sometimes feel compelled to ‘protect’ parents from researchers to decrease the demands on them.

### Data collection strategies must not increase parental burden

Most parents would prefer telephone and/or internet-based data collection strategies as opposed to paper questionnaires or diaries. This is particularly important for parents who are experiencing emotional and psychological distress related to stress/trauma.

### Flexibility and control

Parents need researchers to be flexible in booking interviews and to work around their schedules

## Author details

Dr Lasiuk is an Assistant Professor in the Faculty of Nursing, University of Alberta and a Certified Mental Health/Psychiatric Nurse (C); her research and clinical interests relate to perinatal mental health. Thea Comeau BA, MEd is a PhD student in Counselling Psychology at McGill University, where her research focuses include wellness in helping professionals, burnout, and valuing in Acceptance and Commitment Therapy.

## Authors' contributions

GL conceived and designed the study; supervised its coordination; participated in data collection and the analysis; and prepared the first draft of the manuscript. TC coordinated all aspects of the study including recruitment, data collection, analysis, and interpretation. All authors read and approved the final manuscript.

## Competing interests

The authors declare that they have no competing interests to declare in relation to this manuscript.

## Supplementary Material

Additional File 1 Click here for file

## Declarations

This article has been published as part of *BMC Pregnancy and Childbirth* Volume 13 Supplement 1, 2013: Preterm Birth: Interdisciplinary Research from the Preterm Birth and Healthy Outcomes Team (PreHOT). The full contents of the supplement are available online at http://www.biomedcentral.com/bmcpregnancychildbirth/supplements/13/S1.

All of the publication fees will be funded by the Preterm Birth and Healthy Outcomes Team Interdisciplinary Team Grant (#200700595) from Alberta Innovates - Health Solutions, formerly the Alberta Heritage Foundation for Medical Research.
