# Supplementary material for: Unexpected: an interpretive description of parental traumas’ associated with preterm birth
Source: BMC Pregnancy Childbirth. 2013 Jan 31;13(Suppl 1):S13. doi: 10.1186/1471-2393-13-S1-S13 (PMC3561145; doi:10.1186/1471-2393-13-S1-S13)
Supplement: Additional File 1 [file 1471-2393-13-S1-S13-S1.pdf]

Interview Guide

We know that having a baby is an important event in every parent's life. The purpose of this study is to understand what it is like for parents and families to have a baby born preterm

There are no 'right' or 'wrong' answers and anything that you are willing to share with me here will be helpful. As we talk, I may ask you for more details, examples, or stories to help me better understand what you are saying. Please let me know if you want to take a break, to stop the interview, or if you would rather not answer a particular question.

NOTE TO INTERVIEWER: Allow the participant ample time to reflect each question and to answer it fully before moving on. Probe for details and encourage them to share examples and stories to illustrate what they are saying.

1. What has it been like for you having an infant/child who was born preterm? [Probes: What has been most surprising to you? What has been most challenging or difficult for you?]

2. What has this experience been like for your family? [What have been the biggest changes? What has been most surprising to you? How has having and caring for a preterm baby affected your family?]

The next few questions will help us understand the different kinds of "costs" that families incur when they have a baby born preterm.

3. What are some of the financial costs associated with having and caring for a preterm (premature) infant/child? [Probes: Before you brought your baby/babies home from the hospital?
   After you brought your baby/babies home from the hospital?]

4. We are hoping to carry out some larger studies to learn more about the different costs of caring for preterm infants/children. What do you think would be the best way for us to collect such cost information from parents over time? For example: ask parents to keep a diary/record of all costs over a period of time (6 months) or a monthly telephone with each parent to ask about costs that the family has experienced?

5. Do you have other suggestions?
